# Clinical Characteristics and Endoscopic Management of Duodenal Lipomas: 10-Year Experience from a Tertiary Center in China

**DOI:** 10.5152/tjg.2023.22617

**Published:** 2023-07-01

**Authors:** Jingyuan Xiang, Ningli Chai, Longsong Li, Ning Xu, Pengju Wang, Yaxuan Cheng, Enqiang Linghu

**Affiliations:** 1 Department of Gastroenterology, The First Medical Center of Chinese PLA General Hospital, Beijing, China; 2 Medical School of Chinese People’s Liberation Army, Beijing, China

**Keywords:** Duodenal lipoma, endoscopy, endoscopic ultrasound, endoscopic resection

## Abstract

**Background/Aims:**

Duodenal lipomas are rarely found in the gastrointestinal tract. Most published literature referring to the tumors is limited to case series. There remained issues about the understanding and management of duodenal lipomas to be clarified. We aimed to investigate the clinical and endoscopic features of duodenal lipomas. Additionally, outcomes of endoscopic resection for duodenal lipomas were evaluated.

**Materials and Methods:**

A total of 29 duodenal lipomas resected endoscopically from December 2011 to October 2021 were included. Clinical characteristics, endoscopic features, and endoscopic ultrasound findings were analyzed retrospectively. The endoscopic resection was performed in 3 ways: hot snare polypectomy, endoscopic mucosa resection, and endoscopic submucosal dissection.

**Results:**

Of the 29 duodenal lipomas, 21 were located at the second portion with a mean size of 25.8 mm (range, 7-60 mm). Yamada type IV was the most common macroscopic type in 14 lesions, exhibiting a tendency of forming large peduncles. Seven patients had digestive symptoms. The occurrence of symptoms is associated with the tumor size. Endoscopic ultrasound was performed on 23 duodenal lipomas, of which 20 demonstrated homogenous echogenicity and 3 presented heterogeneous with tubular anechoic region. The endoscopic resection operation was successfully conducted on 29 patients without severe adverse events. The rate of en bloc and endoscopic complete resection was 93.1% and 86.2%, respectively. Recurrence was noted in 1 patient.

**Conclusion:**

Clinical characteristics with typical endoscopic ultrasound features are helpful in duodenal lipomas diagnosis. The endoscopic resection is a safe and effective treatment for duodenal lipomas with considerable long-term outcomes.

**Main Points**

- Lipomas are extremely rare in the duodenum and related studies focusing on the diagnosis and management are limited.
- Yamada type IV is the most common macroscopic type in duodenal lipomas, demonstrating a tendency of forming peduncles.
- Typical endoscopic ultrasound imaging contributes to the diagnosis of duodenal lipomas.
- Endoscopic resection is safe and effective in managing duodenal lipomas with considerable outcomes.

## Introduction

Gastrointestinal (GI) lipomas are uncommonly found in the digestive tract. The majority of GI lipomas occur in the colon, followed by the ileum and jejunum, while duodenal lipomas (DLs) are extremely rare.^1^ The DLs are usually benign and asymptomatic, detected incidentally by computed tomography (CT) or endoscopic examination. However, several reports of acute bleeding, intussusception, and obstruction caused by DLs have been published previously.^2–4^ There are available cases of DLs coexistent with cancerous lesions.^5^ It seems difficult to make a differential diagnosis from other submucosal tumors (SMTs) because DLs are always overlying with GI mucosa. Endoscopic ultrasound (EUS) and CT are reported to be useful in diagnosis, while there is a lack of sufficient research to evaluate current diagnostic methods systematically.^6,7^ Thus, a better understanding of DLs is needed in medical practice.

Endoscopic surveillance is recommended for asymptomatic DLs in consideration of low malignancy.^8^ But, this might result in delayed treatments, especially in the DLs with a growing tendency. Moreover, the continuous follow-up strategy would bring about an extra medical and psychological burden, significantly impairing the patient’s quality of life.

The standard treatment and intervention timing for DLs removal have not been well established. Surgical excision is the prior method for large tumors or emergent circumstances caused by DLs. However, surgical approaches are generally invasive and associated with severe complications.^9-11^ With the advent of advanced facilities and operative techniques, endoscopic resection (ER) has become an alternative treatment for DLs.^12^

Due to the low incidence, only few studies have ever focused on the clinical characteristics and management of DLs, most of which are limited to case series. In the present study, we described and analyzed the clinical features of DLs based on our 10-year experience. The safety and efficacy of ER for DLs were evaluated in the meantime.

## Materials and Methods

The study was retrospectively carried out in the First Medical Center of Chinese PLA General Hospital. From December 2011 to October 2021, a consecutive of 29 patients diagnosed with DLs were registered at our center. All patients underwent ER to remove the tumors. Patients’ baseline data, endoscopic characteristics of the DLs, and outcomes of ER were collected and analyzed.

The indications for ER were as follows: (i) tumor size ≥ 20 mm in diameter, (ii) occurrence of digestive symptoms, (iii) tumor with growing tendency and difficulty to diagnose, and (iv) patients with a desire for ER treatment. The study was approved by the Chinese PLA General Hospital’s Ethics Committee, and written informed consent was obtained from all the patients.

### Endoscopic Management

**Figure 1.**
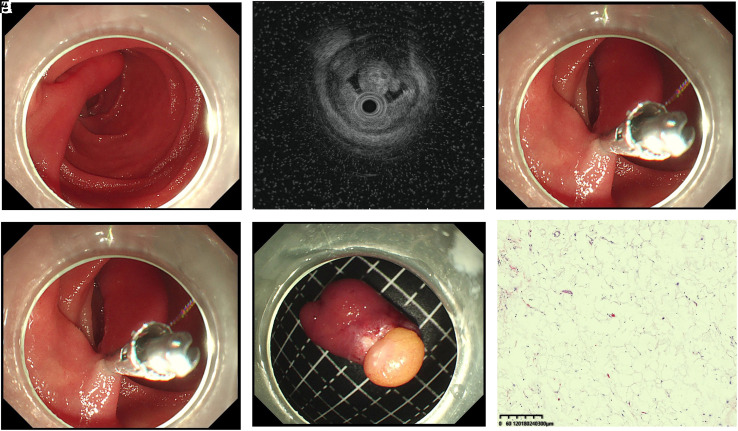
Endoscopic resection of DL by hot snare polypectomy (HSP). (A) A polypoid mass with long peduncle was shown at the descending duodenum. (B) The EUS revealed a hyperechoic lesion with significant echo attenuation beyond the tumor. (C) A clip was used to ligate the tumor base. (D) The tumor was removed with hot snare. (E) Resected specimen with yellow fat tissue. (F) Histopathological results confirmed the tumor was composed of adipose cell. EUS, endoscopic ultrasound; DL, duodenal lipoma.

**Figure 2.**
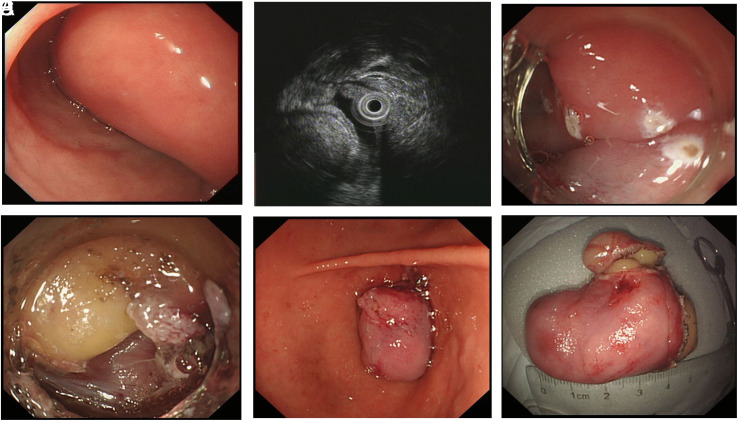
Endoscopic submucosal dissection of large DL. (A) A Yamada type II tumor with broad base was present. (B) Echo attenuation was observed both inside and behind the tumor on EUS. The posterior margin of the tumor was obscure. (C) The lesion was marked by circumferential dots. (D) After the submucosal injection, the lesion was gradually resected by a dual knife. (E) Extraction of the lesion. (F) The specimen was about approximately 5×3 cm. EUS, endoscopic ultrasound; DL, duodenal lipoma.

The EUS was conducted with an ultrasonic probe (Olympus, Tokyo, Japan), investigating the originating layer and growing pattern of the tumors. During the operation procedure, 3 ER techniques were used: hot snare polypectomy (HSP), endoscopic mucosa resection (EMR), and endoscopic submucosal dissection (ESD). Generally speaking, HSP was adopted for protruding tumors with long and thick peduncles. In HSP, the base of the lesion was tightened by an endoloop or a metal clip with the aim of wound closure. Then, the lesion was resected with a snare cautery (Figure 1). The EMR and ESD were adopted for the sessile lesions with a broad base. In EMR, a mixed solution of normal saline, epinephrine (1:10,000), and indigo carmine dye were injected into the submucosal layer to lift the tumors away from the muscularis propria (MP) layer. The tumors were then resected directly with a hot snare. In ESD, the tumor was first marked by circumferential dots with a dual knife (Olympus). Then, the injection step was performed in the same way as in the EMR procedure. Gradually dissecting in the submucosa layer, the tumors were removed away completely with a dual knife or IT knife (Olympus) (Figure 2).

### Histopathological Examination

All the resected specimens were fixed by 10% neutral-buffered formalin, embedded in paraffin wax, and sliced into 5-mm sections. Tissue slices were reviewed after hematoxylin and eosin staining.

### Follow-Up

An abdominal x-ray or CT was performed to monitor delayed perforation. All patients were kept fasting and proton pump inhibitor was routinely administered at the first 3 days after ER treatment. Follow-up endoscopy was arranged at 6 months, then yearly thereafter or whenever patients had complaints of related digestive symptoms.

### Definitions and Outcomes

En bloc was achieved if the tumor was resected as a single piece instead of multiple segments. Endoscopic complete resection was defined as en bloc of the tumor without residual lesion under endoscopy. Severe complications were defined as perforation, massive bleeding, and other adverse events, which might need surgical interventions or prolong the hospital stay. The macroscopic appearance of the DLs was described by Yamada classification: type I, elevations with a smooth baseline without a clear boundary; type II, elevations with a boundary at the base but no notch; type III, elevations with a clearly notched base but no peduncle; and type IV, pedunculated elevations.^13^

### Statistical Analysis

Statistical analysis was performed using Statistical Package for the Social Sciences software (version 25; IBM corp., Armonk, NY, USA). Quantitative data were expressed as the means ± standard deviations or medians with ranges and calculated by Student’s *t*-test. Categorical data were presented as proportions and assessed by the chi-square test or Fisher’s exact test. A *P* value < .05 was considered statistically significant.

## Results

### Baseline and Clinical Data

In total, 29 patients were included in our study. The baseline data are summarized in Table 1. There were 14 men and 15 women, with a mean age of 60.9 years (range, 34-83 years). All tumors were solitary. The mean size was 25.8 mm (range, 7-60 mm). The majority of the tumors (21/29, 72.4%) were located at the second portion of the duodenum, and 8 were located at the bulb portion. Yamada type IV was the most common macroscopic appearance in 14 patients, followed by type II (9, 31.0%), type III (5, 17.3%), and type I (1, 3.4%). Twenty-five lesions (86.2%) showed positive cushion signs when pressed by biopsy forceps. Local erosion or ulcer was noted in 5 lesions.

Twenty-two lesions were silent without clinical symptoms, incidentally discovered during endoscopy or CT examination. Of the remaining 7 patients, 4 patients were accompanied by nonspecific epigastric pain, 1 patient with abdominal distension, 1 patient with melena, and 1 patient with occult blood in feces. The occurrence of melena was seen in a 69-year-old female. Laboratory examination showed the hemoglobin level was 8.2 g/dL before ER treatment. Under endoscopy, a finger-like, Yamada type IV tumor with a thick peduncle was revealed at the second portion. The tumor was approximately 30 mm in length. Deep ulcers and oozing blood could be observed on the surface. The ESD was then performed to remove the tumor completely. The postoperation hemoglobin level gradually rose to the normal range during the follow-up period.

### Endoscopic Ultrasound Features of Duodenal Lipomas

The EUS was performed in 23 (79.3%) patients (Table 2). All tumors presented as hyperechoic masses with intraluminal growing patterns and originated from submucosa (the third layer). A distinct margin could be observed between the tumor body and adjacent tissue in 18 patients. The posterior border was obscure to recognize in 5 patients, due to the significant echo attenuation. Of the 23 DLs, 20 lesions demonstrated homogenous echogenicity. The other 3 demonstrated heterogeneous echogenicity with the internal tubular anechoic region. Echo attenuation beyond the tumors was seen in 18 patients and both inside and beyond the tumors in 5 patients.

### Outcomes of Endoscopic Treatment

The ER operation was successfully conducted on 29 patients. Table 3 shows the clinical outcomes of ER for DLs. The HSP was performed on 16 patients, EMR was performed on 9 patients, and ESD was performed on 4 patients. The median operation time was 13.0 minutes (range, 5-34 minutes) and the mean hospital stay was 9.9 days (range, 6-17 days). En bloc resection was achieved in 27 patients (93.1%). Two lesions were resected piecemeal because of the large size and involvement with the deeper MP layer. Lesion residue with yellowish fatty tissue was identified in 2 patients, who were treated by HSP. In total, the endoscopic complete resection rate was 86.2% (25/29). No procedure-related severe complications were observed, except for 3 patients who had complaints of mild epigastric pain on the first day after the operation. Conservative treatments were adopted and the symptoms were relieved 2-3 days later without any adverse events occurrence.

### Follow-Up

In our study, the median follow-up period was 50 months (8-128 months). The majority of symptomatic patients experienced significant relief after ER treatment. No tumor recurrence was noted in endoscopic complete resection cases. Among the 4 patients with incomplete resection, the recurrent lesion was noted in 1 patient who received HSP treatment. Additional EMR was then performed and a histopathological examination confirmed the diagnosis of lipoma. The patient was kept followed up for 16 months without tumor recurrence.

### Comparisons of the Patients With and Without Clinical Symptoms

To evaluate risk factors associated with clinical symptoms, the patients were divided into a symptomatic group and an asymptomatic group. Clinical baseline data are summarized and compared in Table 4. The analysis revealed that age, gender, tumor location, and macroscopic appearance were comparable between the 2 groups. The occurrence of clinical symptoms was significantly associated with the tumor size (*P* = .028).

## Discussion

Lipomas are rarely found in the GI tract, especially in the duodenum. The DLs accounted for only 0.16% of all GI tumors.^14^ The exact etiology of DLs remains unclear. It is supposed that the tumors might be associated with a disorder of fat metabolism or displacement of the adipose cell.^15^ Till now, the number of clinical studies referring to the DLs’ characteristics and endoscopic management is limited. In a retrospective study, the efficacy of ER for GI lipomas located from the gastric body to the rectum has been identified by Lee et al.^16^ Nevertheless, endoscopic operations in the duodenum are quite different from other GI segments. The duodenal space is narrow and the intestinal wall is thin, leading to technical difficulty and increasing the risk of operation-related complications. To the best of our knowledge, this is the largest-cohort clinical research solely focusing on the endoscopic features and treatments of DLs.

The DLs usually occur in middle age without significant gender predominance.^17^ The tumors are generally slow-growing, benign, and asymptomatic, mostly arising from the descending part of the duodenum.^18^ Large tumors could prolapse into the distal intestinal lumen or proximal gastric cavity, thus resulting in various symptoms. There are several pieces of literature reporting acute intussusception, obstruction, or GI bleeding caused by large DLs. ^2–4,19^ However, the association between tumor size and clinical symptoms is suspected mainly based on previous case reports and individual experience. Few studies have been conducted to evaluate the risk factors of symptomatic DLs. In our study, 7 patients had complaints of digestive symptoms. Clinical characteristics of the symptomatic and asymptomatic DLs were compared. Our analysis showed that tumor size in the symptomatic group was significantly larger than that of the asymptomatic group, consistent with previous findings. Apart from lesion-related symptoms, Iwatsubo et al^5^ has ever introduced a case of DL covered by a duodenal tubular adenoma, suggesting the possibility of a synchronous tumor in DLs. Thus, though DLs generally demonstrate a low risk of malignant change, the accompanying complications deserve enough attention.

Because DLs are composed of mature fat tissue, the tumors could be manifested with a yellowish hue or “cushion sign” when probed by biopsy forceps. However, it remains difficult to distinguish DLs from other SMTs or intestinal polyps just from macroscopic appearance under endoscopy. The EUS has been considered to be a helpful modality in aiding diagnosis. On EUS images, the DLs are typically presented as homogeneous, hyperechoic lesions originating from the submucosal layer.^7^ Occasionally, the tumors could be heterogeneous with scattered calcification or blood vessels inside the tumor body. In our study, 3 cases were observed with tubular anechoic structures as blood vessels sign. Furthermore, the echo attenuation should be noticed. Theoretically speaking, DLs could be diagnosed by the featured echo attenuation, similar to the ultrasonic appearance of fatty liver. But the originating layer of tumors would become obscure and ultrasonic characteristics of deeper structure might be misunderstood under this circumstance. For those tumors located in deep, the involvement with MP layers should be assessed carefully by EUS to guide subsequent treatment choices.

Homogeneous, hyperechoic mass originating from the submucosal layer is not always indicating the DLs. Similar ultrasonographic findings could be observed in other duodenal subepithelial lesions. The most common disease that needs to be differentiated is Brunner’s gland hamartoma. Besides, Figueiredo et al^20^ also reported a rare case of duodenal subepithelial lesion located at the second portion. The EUS showed the lesion was located in the submucosa, demonstrating slightly hyperechoic. To make a further diagnosis, fine-needle aspiration was performed and the histopathological examination revealed the outcome of gangliocytic paraganglioma. Computed tomography is another effective method for DL diagnosis. Instead of echo intensity on EUS, CT could provide the definite value of the tumors, which is more objective and accurate. Hu et al^6^ performed a retrospective analysis of DLs which were diagnosed by CT and found CT value of DLs ranged from −106 to −40 HU, consistent with the fatty density.

Traditionally, DLs are managed by surgical approaches, including segmental resection and pancreaticoduodenectomy. In recent years, ER has become an alternative treatment for duodenal subepithelial tumors with minimal invasion.^8,12,15^ The ER could achieve the goal of removing lesions safely and maintaining the organ’s integrity. Nevertheless, ER in the duodenum is technically difficult owing to its anatomical characteristics. In addition, the postoperation ulcer is directly exposed to the bile and pancreatic juice with rich digestive enzymes, which would aggravate inflammation reaction at the local resection site. Thus, ER treatment is associated with a high risk of intra-operation and delayed adverse events. In consideration of the benign characteristics of lipomas, the treatment strategy of DLs and the necessity of ER operation should be comprehensively evaluated based on the patients’ personal condition, especially in asymptomatic cases.

There is still a lack of universal consensus on the optimal timing and endoscopic method to remove GI lipomas.^21–23^ Tumors ≥2 cm or accompanied by symptoms are generally considered good candidates for ER. While for those tumors <2 cm, endoscopic monitoring is recommended. Nevertheless, continuous follow-up would increase the patients’ financial and psychological burden. Some patients would pay excessive attention to the disease and become nervous about the tumor existence. Certain DLs demonstrate the potential of rapidly growing, which could cause acute rupture or severe obstruction in the GI tract. In such conditions, ER might be performed to remove the tumors in the early stage.

The choice of exact ER technique mainly depends on the tumor shape, location depth, and stalk thickness.^24^ The DLs with long and thick stalks indicate tight involvement with the muscular layer. To manage these lesions, an endoloop or a metal clip is usually used to ligate the tumors’ stalk base. Then, the tumor is removed by a hot snare. The EMR and ESD are performed in the sessile lesions for complete resection. Our study suggested all 3 endoscopic techniques could achieve considerable outcomes in treating DLs. Compared with EMR and ESD, HSP is easier to conduct and could shorten the operation time. Nevertheless, insufficient snare would result in tumor residue and recurrence, as the case in our study.

There are several limitations of our study. First, this was a retrospective study from a single center. Though the time span is as long as 10 years, the sample size is small and selection bias might exist. Prospective studies with large cohorts are needed to further prove the results. Second, ER operation was performed by 2 experienced endoscopists in our study. Thus, no severe adverse events were observed. The outcomes might be a lack of universal generality in other centers. Third, 3 endoscopic interventions were taken as a whole for analysis. Of the 29 patients, more than half underwent HSP treatment and insufficient evaluation for EMR and ESD might be caused. But this could be explained by the fact that Yamada type IV was the most common gross type in our study, which is more suitable for HSP treatment. The comparative outcomes between different endoscopic techniques should be investigated in the next trial.

## Conclusion

In conclusion, DLs are rare tumors in the GI tract. The endoscopic morphology of DLs is diverse. Yamada type IV is the most common macroscopic type and the tumors demonstrate a tendency of forming peduncles. The occurrence of clinical symptoms is associated with tumor size. The DLs exhibit a hyperechoic SMT with distinct margins on EUS image. The EUS has a diagnostic value combined with typical endoscopic findings. The ER is safe and effective in managing DLs with considerable outcomes.

## Figures and Tables

**Table 1.** Clinical and Endoscopic Characteristics of 29 DLs in 29 Patients

Characteristics	
Age, years, mean (range)	60.9 (34-83)
Gender	
Male	14 (48.3)
Female	15 (51.7)
Symptoms, n (%)	
Epigastric pain	4 (13.8)
Abdominal distension	1 (3.4)
Melena	1 (3.4)
Occult blood in feces	1 (3.4)
None	22 (76.0)
Lesions	
Lesion size, mm, mean (range)	25.8 (7-60)
Lesion location, n (%)	
First portion	8 (27.6)
Second portion	21 (72.4)
Macroscopic appearance, n (%)	
Type I	1 (3.4)
Type II	9 (31.0)
Type III	5 (17.3)
Type IV	14 (48.3)
Cushion sign (+), n (%)	25 (86.2)

DLs, duodenal lipomas.

**Table 2.** EUS Features of 23 DLs

EUS Features	Value
Hyperechoic, n (%)	23 (100)
Border	
Distinct margin, n (%)	18 (78.3)
Indistinct margin, n (%)	5 (21.7)
Originating layer	
Mucosa, n (%)	0 (0)
Submucosa, n (%)	23 (100)
Echogenicity	
Homogenous, n (%)	20 (87.0)
Heterogeneous, n (%)	3 (13.0)
Echo attenuation	
Beyond, n (%)	18 (78.3)
Inside and beyond, n (%)	5 (21.7)

DLs, duodenal lipomas; EUS, endoscopic ultrasound.

**Table 3.** Therapeutic Outcomes of Endoscopic Treatments

Outcomes	Value
Endoscopic treatment, n (%)	
HSP	16 (55.2)
EMR	9 (31.0)
ESD	4 (13.8)
Success rate, % (n)	100 (29)
Operation time, minutes, median (range)	13 (5-34)
En bloc resection, n (%)	27 (93.1)
Endoscopic complete resection, n (%)	25 (86.2)
Complications	
Perforation, n (%)	0 (0)
Massive bleeding, n (%)	0 (0)
Epigastric pain, n (%)	3 (10.3)
Hospital stay, days, mean (range)	9.9 (6-17)
Follow-up, months, median (range)	50 (8-128)
Recurrence rate, % (n)	3.4 (1)

EMR, endoscopic mucosa resection; ESD, endoscopic submucosal dissection; HSP, hot snare polypectomy.

**Table 4.** Comparisons of the Patients With and Without Clinical Symptoms

	With Symptoms (n = 7)	Without Symptoms (n = 22)	*P*
Age, years, mean	66.4 ± 7.1	59.2 ± 12.9	.170
Gender			.231
Male	2	12	
Female	5	10	
Lesion location, n (%)			.299
Bulb	3	5	
Second portion	4	17	
Lesion size, mm, n (%)			.028
<2 cm	0	10	
≥2 cm	7	12	
Macroscopic appearance			.912
Type I	0	1	
Type II	2	7	
Type III	1	4	
Type IV	4	10	
